# Obesity is a strong risk factor for short-term mortality and adverse outcomes in Mexican patients with COVID-19: a national observational study

**DOI:** 10.1017/S0950268821001023

**Published:** 2021-04-29

**Authors:** J. M. Vera-Zertuche, J. Mancilla-Galindo, M. Tlalpa-Prisco, P. Aguilar-Alonso, M. M. Aguirre-García, O. Segura-Badilla, M. Lazcano-Hernández, H. I. Rocha-González, A. R. Navarro-Cruz, A. Kammar-García, J. de J. Vidal-Mayo

**Affiliations:** 1 Endocrinology Department, Obesity Clinic, Instituto Nacional de Ciencias Médicas y Nutrición Salvador Zubirán, Mexico City, Mexico; 2 Unidad de Investigación UNAM-INC, Instituto Nacional de Cardiología Ignacio Chávez, Mexico City, Mexico; 3 Sección de Estudios de Posgrado e Investigación, Escuela Superior de Medicina, Instituto Politécnico Nacional, Mexico City, Mexico; 4 Facultad de Ciencias Químicas, Departamento de Bioquímica y Alimentos, Benemérita Universidad Autónoma de Puebla, Puebla, Mexico; 5 Facultad de Ciencias de la Salud y de los Alimentos, Departamento de Nutrición y Salud Pública, Programa UBB Saludable, Universidad del Bío-Bío, Bío-Bío, Chile; 6 Emergency Department, Instituto Nacional de Ciencias Médicas y Nutrición Salvador Zubirán, Mexico City, Mexico

**Keywords:** Comorbidities, coronavirus, COVID-19, mortality, obesity, SARS-CoV-2

## Abstract

Conflicting results have been obtained through meta-analyses for the role of obesity as a risk factor for adverse outcomes in patients with coronavirus disease-2019 (COVID-19), possibly due to the inclusion of predominantly multimorbid patients with severe COVID-19. Here, we aimed to study obesity alone or in combination with other comorbidities as a risk factor for short-term all-cause mortality and other adverse outcomes in Mexican patients evaluated for suspected COVID-19 in ambulatory units and hospitals in Mexico. We performed a retrospective observational analysis in a national cohort of 71 103 patients from all 32 states of Mexico from the National COVID-19 Epidemiological Surveillance Study. Two statistical models were applied through Cox regression to create survival models and logistic regression models to determine risk of death, hospitalisation, invasive mechanical ventilation, pneumonia and admission to an intensive care unit, conferred by obesity and other comorbidities (diabetes mellitus (DM), chronic obstructive pulmonary disease, asthma, immunosuppression, hypertension, cardiovascular disease and chronic kidney disease). Models were adjusted for other risk factors. From 24 February to 26 April 2020, 71 103 patients were evaluated for suspected COVID-19; 15 529 (21.8%) had a positive test for SARS-CoV-2; 46 960 (66.1%), negative and 8614 (12.1%), pending results. Obesity alone increased adjusted mortality risk in positive patients (hazard ratio (HR) = 2.7, 95% confidence interval (CI) 2.04–2.98), but not in negative and pending-result patients. Obesity combined with other comorbidities further increased risk of death (DM: HR = 2.79, 95% CI 2.04–3.80; immunosuppression: HR = 5.06, 95% CI 2.26–11.41; hypertension: HR = 2.30, 95% CI 1.77–3.01) and other adverse outcomes. In conclusion, obesity is a strong risk factor for short-term mortality and critical illness in Mexican patients with COVID-19; risk increases when obesity is present with other comorbidities.

## Introduction

The severe acute respiratory syndrome coronavirus 2 (SARS-CoV-2) causes an acute respiratory and systemic disease known as coronavirus disease (COVID-19). Increasing number of studies have linked obesity with adverse outcomes in patients who develop COVID-19 [1]. Systemic adiposity at all levels (subcutaneous, visceral and ectopic) may complicate the management of acute lung and systemic diseases such as COVID-19 through mechanical and inflammatory complications that commonly occur in the ‘chronic diseases associated with adiposity’ [2].

Mechanical consequences derive from the accumulation of adipose tissue in soft tissues of the pharynx, which may compromise ventilation, and the visceral compartment at the mesenteric and omental levels, causing renal compression that favours systemic arterial hypertension and other dysregulations [3]. Inflammatory mechanisms are promoted when adipocytes saturate their storage capacity and undergo apoptosis, leading to a local inflammatory response that favours remodelling of adipose tissue with a phenotypic switch of regulatory M2 macrophages to pro-inflammatory M1 macrophages which release pro-inflammatory cytokines (i.e. interleukin (IL)-1*β*, TNF-*α*, IL-6) at the systemic level [4]. The chronic pro-inflammatory state that characterises obesity could contribute to disease progression in COVID-19 due to a strong, uncontrolled and sustained systemic inflammatory response.

The Mexican population is metabolically different from people of Asian and European descent, having a higher prevalence of early-onset overweight and obesity [5]. Chronic overfeeding in quantity and frequency of highly processed foods has unleashed an epidemic of obesity in recent decades [6]. Currently, 75.2% of the Mexican population over 20 years of age is overweight (39.1%) or obese (36.1%) [7]; thus, the toll of COVID-19 could be higher in Mexico by affecting younger people compared to populations with a lower burden of disease among young adults.

Increased age, chronic obstructive pulmonary disease (COPD), cardiovascular disease (CVD), diabetes mellitus (DM), obesity, chronic kidney disease (CKD) and immunocompromise have been established as the main risk factors for severe disease in Mexican patients with COVID-19 [8–10]. Obesity is the comorbidity with the strongest association with a positive test for SARS-CoV-2 in Mexican patients with predominantly symptomatic COVID-19 [11]. However, the role of obesity as a risk factor in COVID-19 remains controversial since one systematic review found that obesity was not a strong predictor for COVID-19 severity and was not associated with increased mortality risk [12], whereas another systematic review found that obesity was associated with increased mortality only in studies with fewer chronic and critical patients [13], suggesting that failure to include patients within the whole spectrum of COVID-19 severity and with fewer comorbidities could mask the risk attributable to obesity.

The impact of obesity as a contributing risk factor has been assessed in studies including predominantly old multimorbid individuals, but not when obesity is the sole or only concomitant comorbidity. This is problematic since estimated risks in multimorbid patients with COVID-19 could be attributable to complex interactions between distinct concomitant diseases and increased age. Therefore, it is important to elucidate if individual comorbidities present in predominantly young patients act as significant risk factors for adverse COVID-19-related outcomes and to explore the effect of their combination with other individual comorbidities.

In this study, we aimed to elucidate if obesity is an independent risk factor for short-term mortality and other adverse outcomes in patients with obesity as their only comorbidity and patients with obesity plus one other comorbidity who were evaluated for suspected COVID-19 in both ambulatory units and hospitals in Mexico.

## Methods

### Study design

We carried out a retrospective observational study in a national cohort of individuals included in Mexico's COVID-19 National Epidemiologic Surveillance Study, in COVID-19-accredited medical units across the national territory of Mexico. To limit variations in testing, mortality rates, changes in standards of care and to avoid the effect of saturation of hospitals, we contemplated 71 103 patients evaluated for suspected COVID-19 in the first 2-month period of the pandemic in Mexico between 24 February and 26 April 2020. Patients were grouped according to SARS-CoV-2 reverse-transcriptase polymerase chain reaction (RT-PCR) result into positive, negative or pending.

### Exposures and outcomes

Patients with obesity as their unique comorbidity, and patients with obesity plus one other comorbidity who were suspected of having COVID-19 were considered as the exposures of interest. The main outcome of interest was all-cause mortality, defined as the occurrence of death up to 56 days after inclusion. The secondary outcomes of interest were the occurrence of hospitalisation, invasive mechanical ventilation (IMV), pneumonia or admission to an intensive care unit (ICU).

### Source of data

We used an open dataset made available through the Directorate General of Epidemiology of Mexico's Secretariat of Health [14], in their Historical Dataset Repository [15]. Criteria for suspected COVID-19 case were at least two of three signs/symptoms (cough, fever or headache) plus at least one other (dyspnoea, arthralgias, myalgias, sore throat, rhinorrhoea, conjunctivitis or chest pain) in the last 7 days.

Clinicians and epidemiologists collect baseline demographic and clinical variables, as well as follow-up variables daily for at least 7 days in ambulatory patients who are considered recovered after 14 days from symptom onset if alive and not hospitalised. For hospitalised patients, follow-up occurs daily until discharge. In order to upload testing results, laboratories are required to have accredited SARS-CoV-2 RT-PCR procedures by the Mexican Institute of Diagnostics and Epidemiological Reference.

Clinical, demographic and follow-up data collected and publicly released were: age, origin, sex, nationality, pregnancy, smoker status, symptom onset date, date of medical attention, contact with another confirmed case, comorbidities (DM, COPD, asthma, immunosuppression, hypertension, CVD, obesity (defined as a body mass index (BMI) ⩾95th percentile for age and sex in patients younger than 18 years, and as a BMI *P* ⩾ 30 in patients 18 years or older) [16] and CKD), clinical diagnosis of pneumonia, IMV and admission to an ICU.

### Management of variables

Age was categorised into six groups: ⩽20 years, 21–30 years, 31–40 years, 41–50 years, 51–60 years and >60 years. Days elapsed from symptom onset to initial care, and days from symptom onset to death were calculated.

Comorbidities were determined through anamnesis during the initial medical evaluation in ambulatory units or hospitals, and classified as dummy variables (present or absent). Patients with the same individual comorbidity were grouped to evaluate impact of isolated comorbidities; those with obesity and only one other comorbidity were also grouped and compared to patients with no comorbidities.

The primary endpoint was all-cause mortality. Secondary endpoints were the hospitalisation, pneumonia, IMV and ICU admission.

Socio-demographic characterisation of the population was performed according to poverty and social lag indicators for every municipality in which people who were suspected of having COVID-19 lived at the moment of inclusion in the study. The socio-demographic variables obtained were social lag index (SLI), ageing index, afro-descendants per 100 inhabitants, indigenous language-speaking per 100 inhabitants, affiliation to health services per 100 inhabitants, hospital evaluating rooms per 10 000 inhabitants, hospital beds per 10 000 inhabitants and members per household [17–19].

The SLI is a pondered measure derived from a principal component analysis of 11 variables that are determinants of education, health, basic urbanisation services and household spaces [18]. The ageing index is the estimated population >65 years divided by those 15 years or younger, and multiplied by 100 [19].

### Statistical analysis

Categorical variables are presented as frequencies and percentages; quantitative variables, as mean and standard deviation (s.d.) or median with interquartile range (IQR). Comparisons between categorical variables were performed with *χ*^2^ tests. For quantitative variables, with Student's *t* test, Mann–Whitney *U* ore one-way analysis of variance (ANOVA) with Welch's test and post-hoc analysis by Tukey's test or Games-Howell's test.

Multivariate regression analyses were carried out through Cox proportional-hazards regression models to predict mortality risk in patients with obesity and other comorbidities, according to test result: positive, negative or pending; this model was used since it was considered suitable to the presentation of data for a time-dependent event (mortality) which is a function of survival. Logistic regression models were performed to determine the risk of other adverse events accordingly. Models for each group were adjusted for sex, age, smoker and time from symptom onset to medical care according to a o value <0.1 in the crude model. To control the confounding effect of socio-demographic variables for severe COVID-19 cases [20], all models were adjusted for the previously described socio-demographic variables.

To corroborate robustness of the association between obesity and other comorbidities and mortality in SARS-CoV-2-positive patients, sensitivity analyses were performed; multivariate-adjusted Cox regression models were repeated in three subgroups after excluding hospitalised, intubated and ICU-admitted patients. A *P* < 0.05 was used to define statistical significance. All analyses were performed in SPSS v.21 and GraphPad Prism v.9.0.2.

## Results

A total of 71 103 patients evaluated for suspected COVID-19 were included for analysis, of which 15 529 (21.8%) tested positive for SARS-CoV-2, 46 960 (66.1%) were negative and 8614 (12.1%) had pending results. Patients were residents of 1468 municipalities from all 32 states of Mexico. In total, 20.5% (*n* = 14 590) of all patients were residents of Mexico City and 11.2% (*n* = 8316) from the State of Mexico. There was a higher proportion of men in positive-test and pending-result groups, whereas more women had a negative test. Patients in the positive-test group were older than negative-test and pending-result patients. The proportions of hospitalised patients, clinical diagnosis of pneumonia, IMV and ICU admission were greater in the positive-test group (Table 1).

**Table 1.** Baseline and follow-up characteristics of patients according to RT-PCR for SARS-CoV-2 test result: positive, negative or pending

	Positive *n* = 15 529	Negative *n* = 46 960	Pending-result *n* = 8614	*P* value
Sex				
Women, *n* (%)	6552 (42.2)	25 005 (53.2)	3950 (45.9)	<0.0001
Men, *n* (%)	8977 (57.8)	21 955 (46.8)	4664 (54.1)	
Age, years	46.6 (15.5)	39.6 (17.9)*	43.2 (17.3)*	<0.0001
Age, *n* (%)				
⩽20	372 (2.4)	4858 (10.3)	622 (7.2)	<0.0001
21–30	2029 (13.1)	10 080 (21.5)	1346 (15.6)	
31–40	3408 (21.9)	11 596 (24.7)	2084 (24.2)	
41–50	3717 (23.9)	8816 (18.8)	1794 (20.8)	
51–60	3717 (23.9)	5778 (12.3)	1376 (16)	
>60	3113 (20)	5832 (12.4)	1392 (16.2)	
Smoker, *n* (%)	1374 (8.8)	4835 (10.3)	811 (9.4)	0.009
Pregnancy, *n* (%)	91 (0.6)	649 (1.4)	58 (0.7)	0.006
Contact with confirmed case, *n* (%)	4230 (27.2)	13 273 (28.3)	3251 (37.7)	<0.0001
Comorbidities, *n* (%)				
DM	2831 (18.2)	5163 (11)	1323 (15.4)	<0.0001
COPD	389 (2.5)	1330 (2.8)	214 (2.5)	0.6
Asthma	542 (3.5)	2571 (5.5)	337 (3.9)	<0.0001
Immunosuppression	296 (1.9)	1402 (3)	183 (2.1)	0.004
Hypertension	3370 (21.7)	7553 (15.7)	1611 (18.7)	<0.0001
CVD	453 (2.9)	1610 (3.4)	267 (3.1)	0.1
Obesity	3215 (20.7)	6570 (14)	1532 (17.8)	<0.0001
CKD	359 (2.3)	1130 (2.4)	262 (3)	0.002
Number of comorbidities, *n* (%)				
No comorbidities	8422 (54.2)	30 316 (63.9)	5057 (58.7)	<0.0001
One comorbidity	4073 (26.2)	10 220 (21.8)	2086 (24.2)	
Two comorbidities	2029 (13.1)	4250 (9.1)	959 (11.1)	
⩾3 comorbidities	1005 (6.5)	2474 (5.3)	512 (5.9)	
Time from symptom onset to medical care, days	4 (2–6)	3 (1–5)*	3 (1–5)*	<0.0001
Hospitalisation, *n* (%)	6042 (38.9)	9974 (21.2)	2607 (30.3)	<0.0001
Pneumonia, *n* (%)	4588 (29.5)	6341 (13.5)	1841 (21.4)	<0.0001
IMV, *n* (%)	669 (4.3)	598 (1.3)	197 (2.3)	<0.0001
ICU admission, *n* (%)	676 (4.4)	774 (1.6)	211 (2.4)	<0.0001
Case-fatality rate, *n* (%)	1434 (9.2)	881 (1.9)	137 (1.6)	<0.0001
Socio-geographical variables				
SLI	−1.32 (−1.41 to −1.11)	−1.29 (−1.41 to −1.06)*	−1.29 (−1.39 to −1.02)*	<0.0001
Ageing index	28.7 (21.8–39.6)	28.6 (21.8–38.6)*	29.2 (21.5–38.6)*	<0.0001
Afro-descendant per 100 inhabitants	0.61 (0.07–1.80)	0.23 (0.03–1.39)*	0.35 (0.03–1.74)*	<0.0001
Indigenous language-speaking per 100 inhabitants	1.22 (0.63–1.89)	0.96 (0.00–1.74)*	1.1 (0.00–1.79)*	<0.0001
Affiliation to health services per 100 inhabitants	80.2 (77.6–84.3)	82.6 (78.6–86.0)*	81.3 (77.4–85.5)	<0.0001
Members per household	3.59 (3.45–3.71)	3.66 (3.50–3.80)*	3.68 (3.50–3.84)*	<0.0001
Hospitals per 10 000 inhabitants	3.50 (2.42–5.51)	4.07 (2.31–6.30)*	3.43 (2.02–5.40)*	<0.0001
Hospital beds per 10 000 inhabitants	11.8 (6.9–18.7)	11.8 (6.9–18.5)	11.2 (5.3–17.9)*	<0.0001

CKD, chronic kidney disease; COPD, chronic obstructive pulmonary disease; CVD, cardiovascular disease; DM, diabetes mellitus; ICU, intensive care unit; Immunosupp, immunosuppression; IMV, invasive mechanical ventilation; SLI, social lag index.

Data are presented as mean (s.d.) or median (1Q–3Q).

Comparisons between groups were made by *χ*^2^ or one-way ANOVA.

*Significantly different from positive patients (*P* < 0.05) by post-hoc analysis by Tukey's test or Games-Howell's test.

The case–fatality rate in positive patients (9.2%) was higher than that of negative (1.9%) and pending-result (1.6%) patients. Of hospitalised patients, 21% of positive-test cases died, whereas only 8.3% and 4.6% in negative and pending-result groups died, respectively (*P* < 0.0001).

Comorbidities of non-survivors in the positive-test, negative-test and pending-result groups were, respectively: DM (38.8%, 40.9% and 38.8%), COPD (7.5%, 14.9% and 8.8%), asthma (3.1%, 2.4% and 5.1%), immunosuppression (4.7%, 10.3% and 4.4%), hypertension (43%, 43.1% and 38.7%), CVD (6.3%, 13.4% and 10.2%), obesity (30.5%, 18% and 23.4%) and CKD (6.6%, 13.4% and 12.4%). Ages of non-survivors were similar (*P* = 0.9) in the positive (59.3 years, s.d.: 14.2), negative (57.9, s.d.: 22.4) and pending-result (57.2, s.d.: 17.5) groups. Non-survivors were older than survivors (*P* < 0.0001): positive-test (59.3, s.d.: 14.2 *vs.* 45.3, s.d.: 15.04), negative-test (57.9, s.d.: 22.4 *vs.* 39.2, s.d.: 17.6) and pending-result (57.2, s.d.: 17.5 *vs.* 42.9, s.d.: 17.2). Median time from symptom onset to medical care was similar between survivors and non-survivors in positive cases (4 days, IQR: 2–6 *vs.* 4 days, IQR: 1–6, *P* = 0.4), but not in negative (2 days, IQR: 0–4.5 *vs.* 3 days, IQR: 1–5, *P* < 0.0001) and pending-result (4 days, IQR: 1.5–7 *vs.* 3 days, IQR: 1–5, *P* = 0.002) groups.

Patients with a positive test who died lived in municipalities with greater SLI than those who survived (−1.33, IQR: −1.42 to −1.11 *vs.* −1.26, IQR: −1.38 to −1.02, *P* < 0.0001); patients who survived lived in municipalities with a higher ageing index (29.1, IQR: 21.8–39.6 *vs.* 26.9, IQR: 20.9–35.7, *P* < 0.0001), and more hospitals per 10 000 inhabitants (3.57, IQR: 2.42–5.53, *vs.* 3.21, IQR: 2.42–5.40, *P* = 0.03).

After grouping patients with only one comorbidity and those with obesity plus only one other comorbidity (Supplementary Tables S1–S3), obesity was found to be one of the leading risk factors for death in positive-test patients. In all mortality risk models (Table 2) for individual comorbidities compared with no comorbidities, patients with obesity had higher adjusted mortality risk than those with DM, COPD, asthma, hypertension and CVD. When obesity was present with another comorbidity, the risk was higher.

**Table 2.** Mortality risk of patients according to RT-PCR for SARS-CoV-2 test result: positive, negative or pending

	Positive^a^		Negative^b^		Pending-result^c^	
	Crude model	Adjusted model	Crude model	Adjusted model	Crude model	Adjusted model
Obesity model						
Without comorbidities	Reference					
Obesity alone	2.37 (1.96–2.86)	2.47 (2.04–2.98)	1.18 (0.81–1.73)	1.05 (0.71–1.53)	1.79 (0.89–3.61)	1.73 (0.86–3.48)
DM model						
Without comorbidities	Reference					
DM alone	3.66 (2.99–4.49)	1.91 (1.55–2.35)	5.92 (4.46–7.85)	2.99 (2.22–4.03)	3.17 (1.58–6.38)	1.74 (0.85–3.59)
Obesity alone	2.36 (1.95–2.84)	2.42 (2.01–2.93)	1.18 (0.81–1.73)	1.05 (0.72–1.54)	1.79 (0.89–3.61)	1.71 (0.85–3.44)
DM + obesity	3.88 (2.85–5.29)	2.79 (2.04–3.80)	3.78 (2.07–6.92)	2.33 (1.27–4.29)	1.91 (0.46–7.92)	1.60 (0.38–6.68)
COPD model						
Without comorbidities	Reference					
COPD alone	5.92 (3.64–9.64)	1.72 (1.05–2.84)	11.98 (8.23–17.44)	3.05 (1.97–4.72)	1.95 (0.27–14.24)	0.58 (0.08–4.44)
Obesity alone	2.36 (1.96–2.86)	2.47 (2.04–2.99)	1.18 (0.81–1.73)	1.05 (0.72–1.54)	1.79 (0.89–3.61)	1.74 (0.86–3.49)
COPD + obesity	4.29 (1.60–11.5)	2.42 (0.89–6.53)	5.84 (1.45–23.5)	1.80 (0.44–7.64)	9.86 (1.35–72.01)	5.13 (0.68–38.59)
Asthma model						
Without comorbidities	Reference					
Asthma alone	0.35 (0.13–0.96)	0.63 (0.24–1.70)	0.49 (0.22–1.11)	0.57 (0.25–1.29)	0.83 (0.14–6.05)	1.40 (0.19–10.37)
Obesity alone	2.37 (1.96–2.86)	2.47 (2.04–2.99)	1.18 (0.81–1.72)	1.04 (0.71–1.53)	1.80 (0.89–3.62)	1.72 (0.85–3.46)
Asthma + obesity	1.42 (0.59–3.45)	2.18 (0.90–5.28)	1.63 (0.61–4.39)	1.81 (0.67–4.87)	Not estimable	
Immunosuppression model						
Without comorbidities	Reference					
Immunosupp. alone	3.04 (1.75–5.28)	2.54 (1.46–4.43)	5.96 (4.05–8.77)	5.22 (3.54–7.70)	4.13 (1.27–13.41)	2.71 (0.79–9.27)
Obesity alone	2.36 (1.96–2.86)	2.45 (2.03–2.97)	1.18 (0.81–1.73)	1.05 (0.72–1.54)	1.79 (0.89–3.61)	1.69 (0.84–3.40)
Immunosupp. + obesity	5.62 (2.51–12.6)	5.06 (2.26–11.41)	6.33 (2.03–19.79)	4.64 (1.49–14.55)	37.6 (5.11–276.44)	34.63 (4.48–267.43)
Hypertension model						
Without comorbidities	Reference					
Hypertension alone	2.98 (2.45–3.65)	1.33 (1.08–1.64)	3.31 (2.48–4.41)	1.39 (1.03–1.89)	2.38 (1.19–4.78)	1.15 (0.55–2.39)
Obesity alone	2.37 (1.96–2.86)	2.46 (2.03–2.97)	1.18 (0.81–1.73)	1.04 (0.71–1.53)	1.78 (0.88–3.57)	1.72 (0.85–3.45)
Hypertension + obesity	3.44 (2.64–4.48)	2.30 (1.77–3.01)	2.43 (1.48–3.97)	1.34 (0.82–2.21)	2.25 (0.81–6.31)	1.49 (0.53–4.22)
CVD model						
Without comorbidities	Reference					
CVD alone	3.55 (2.05–6.18)	2.00 (1.15–3.48)	4.75 (2.47–8.14)	2.89 (1.66–5.02)	2.75 (0.95–11.64)	1.57 (0.34–7.21)
Obesity alone	2.37 (1.96–2.86)	2.47 (2.04–2.98)	1.18 (0.81–1.73)	1.05 (0.72–1.53)	1.79 (0.89–3.59)	1.69 (0.84–3.40)
CVD + obesity	2.82 (0.70–11.3)	1.44 (0.36–5.78)	7.82 (2.91–21.02)	4.33 (1.59–11.72)	7.53 (1.03–55.03)	4.87 (0.55–43.34)
CKD model						
Without comorbidities	Reference					
CKD alone	1.77 (0.66–4.76)	1.24 (0.46–3.34)	6.81 (3.51–13.26)	3.55 (1.80–6.98)	8.07 (1.95–33.46)	5.79 (1.34–24.89)
Obesity alone	2.37 (1.96–2.86)	2.47 (2.04–2.98)	1.18 (0.81–1.73)	1.05 (0.72–1.53)	1.79 (0.89–3.61)	1.74 (0.86–3.50)
CKD + obesity	2.69 (0.38–19.2)	4.63 (0.65–33.1)	10.37 (2.57–41.71)	6.24 (1.55–25.19)	15.96 (2.18–116.79)	10.35 (1.39–76.83)

95% CI, 95% confidence interval; CKD, chronic kidney disease; COPD, chronic obstructive pulmonary disease; CVD, cardiovascular disease; DM, diabetes mellitus; HR, hazard ratio; Immunosupp, immunosuppression.

Data are presented as HR (95% CI).

a Model adjusted by sex, age and time from symptom onset to care, SLI, ageing index, afro-descendant per 100 inhabitants, indigenous language-speaking per 100 inhabitants, affiliation to health services per 100 inhabitants, members per household, hospitals per 10 000 inhabitants, hospital beds per 10 000 inhabitants.

b Model adjusted by sex, age and time from symptom onset to care, SLI, ageing index, afro-descendant per 100 inhabitants, indigenous language-speaking per 100 inhabitants, affiliation to health services per 100 inhabitants, members per household, hospitals per 10 000 inhabitants, hospital beds per 10 000 inhabitants.

c Model adjusted by sex, age, smoking and time from symptom onset to care, SLI, ageing index, afro-descendant per 100 inhabitants, indigenous language-speaking per 100 inhabitants, affiliation to health services per 100 inhabitants, members per household, hospitals per 10 000 inhabitants, hospital beds per 10 000 inhabitants.

No changes in the findings of primary models resulted from sensitivity analyses for obesity, but risks for other comorbidities did change (Table 3). An increased risk of death in patients with obesity alone persisted after excluding hospitalised patients, those requiring IMV or admitted to ICU.

**Table 3.** Sensitivity analyses of mortality risk in SARS-CoV-2-positive cases

	Excluding hospitalised patients	Excluding patients requiring IMV	Excluding patients admitted to ICU
Obesity model			
Without comorbidities	Reference		
Obesity alone	3.13 (1.71–5.75)	2.37 (1.89–2.97)	2.50 (2.02–3.09)
DM model			
Without comorbidities	Reference		
DM alone	2.90 (1.53–5.50)	2.17 (1.73–2.74)	2.06 (1.64–2.59)
Obesity alone	2.98 (1.63–5.45)	2.32 (1.85–2.90)	2.46 (1.99–3.05)
DM + obesity	2.04 (0.62–6.73)	2.66 (1.83–3.85)	2.90 (2.05–4.11)
COPD model			
Without comorbidities	Reference		
COPD alone	2.39 (0.51–11.17)	1.66 (0.92–2.99)	1.61 (0.89–2.89)
Obesity alone	3.13 (1.71–5.74)	2.37 (1.89–2.97)	2.52 (2.03–3.12)
COPD + obesity	7.05 (0.93–53.21)	2.25 (0.71–7.08)	1.19 (0.69–6.88)
Asthma model			
Without comorbidities	Reference		
Asthma alone	Not estimable	0.42 (0.10–1.68)	0.20 (0.03–1.40)
Obesity alone	3.13 (1.71–5.75)	2.37 (1.89–2.97)	2.50 (2.02–3.10)
Asthma + obesity	Not estimable	1.71 (0.55–2.97)	1.63 (0.52–5.11)
Immunosuppression model			
Without comorbidities	Reference		
Immunosupp. alone	2.68 (0.36–19.71)	2.65 (1.45–4.87)	2.83 (1.58–5.07)
Obesity alone	3.11 (1.69–5.70)	2.35 (1.88–2.95)	2.49 (2.01–3.08)
Immunosuppression + obesity	Not estimable	6.38 (2.84–14.35)	5.63 (2.32–13.66)
Hypertension model			
Without comorbidities	Reference		
Hypertension alone	1.32 (0.67–2.58)	1.31 (0.03–1.67)	1.39 (1.10–1.76)
Obesity alone	3.17 (1.74–5.80)	2.37 (1.89–2.96)	2.50 (2.02–3.09)
Hypertension + obesity	2.51 (1.05–6.01)	1.99 (1.43–2.77)	2.14 (1.59–2.93)
CVD model			
Without comorbidities	Reference		
CVD alone	Not estimable	1.64 (0.81–3.32)	1.57 (0.78–3.17)
Obesity alone	3.13 (1.71–5.75)	2.38 (1.89–2.97)	2.51 (2.03–3.10)
CVD + obesity	Not estimable	Not estimable	0.81 (0.11–5.77)
CKD model			
Without comorbidities	Reference		
CKD alone	Not estimable	0.92 (0.23–3.3.69)	0.38 (0.05–2.72)
Obesity alone	3.13 (1.71–5.75)	2.37 (1.89–2.97)	2.50 (0.02–3.09)
CKD + obesity	Not estimable	Not estimable	5.65 (0.79–40.52)

95% CI, 95% confidence interval; CKD, chronic kidney disease; COPD, chronic obstructive pulmonary disease; CVD, cardiovascular disease; DM, diabetes mellitus; HR, hazard ratio; ICU, intensive care unit; IMV, invasive mechanical ventilation.

Data presented as HR (95% CI).

All models were adjusted by sex, age, SLI, ageing index, afro-descendant per 100 inhabitants, indigenous language-speaking per 100 inhabitants, affiliation to health services per 100 inhabitants, members per household, hospitals per 10 000 inhabitants, hospital beds per 10 000 inhabitants.

**Fig. 1.**
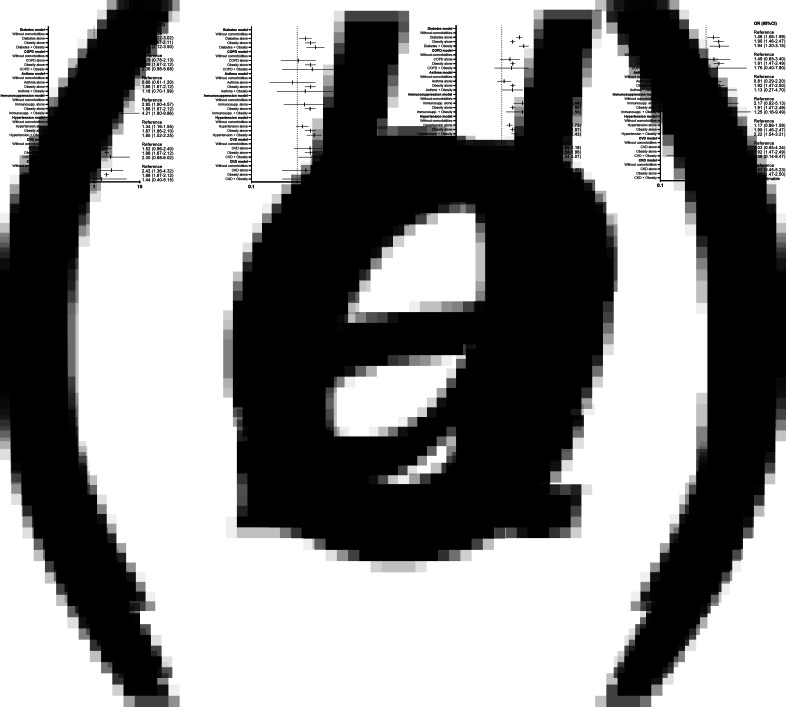
Risk of secondary adverse outcomes in patients with a positive test for SARS-CoV-2 with obesity or obesity plus one other comorbidity. (a) Risk of hospitalisation, model adjusted by sex, age, smoker, SLI, ageing index, afro-descendant per 100 inhabitants, indigenous language-speaking per 100 inhabitants, affiliation to health services per 100 inhabitants, members per household, hospitals per 10 000 inhabitants, hospital beds per 10 000 inhabitants. (b) Risk of IMV, model adjusted by sex, age, smoker and time from symptom onset to care, SLI, ageing index, afro-descendant per 100 inhabitants, indigenous language-speaking per 100 inhabitants, affiliation to health services per 100 inhabitants, members per household, hospitals per 10 000 inhabitants, hospital beds per 10 000 inhabitants. (c) Risk of pneumonia, model adjusted by sex, age, smoker and time from symptom onset to care, SLI, ageing index, afro-descendant per 100 inhabitants, indigenous language-speaking per 100 inhabitants, affiliation to health services per 100 inhabitants, members per household, hospitals per 10 000 inhabitants, hospital beds per 10 000 inhabitants. (d) Risk of admission to ICU, model adjusted by sex, age and time from symptom onset to care, SLI, ageing index, afro-descendant per 100 inhabitants, indigenous language-speaking per 100 inhabitants, affiliation to health services per 100 inhabitants, members per household, hospitals per 10 000 inhabitants, hospital beds per 10 000 inhabitants. CKD, chronic kidney disease; COPD, chronic obstructive pulmonary disease; CVD, cardiovascular disease; Immunosupp, immunosuppression; ICU, intensive care unit; IMV, invasive mechanical ventilation.

**Fig. 2.**
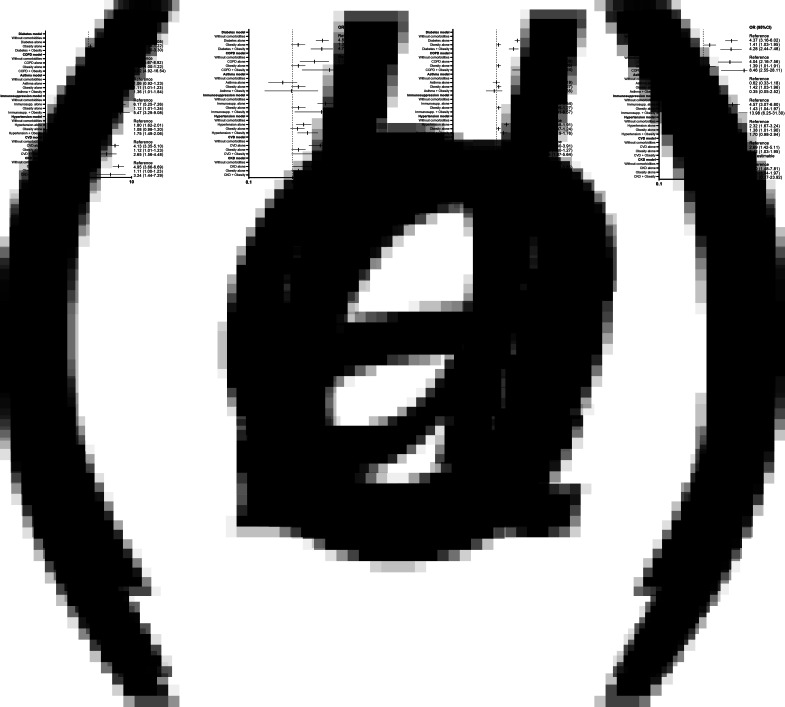
Risk of secondary adverse outcomes in patients with a negative test for SARS-CoV-2 with obesity or obesity plus one other comorbidity. (a) Risk of hospitalisation, model adjusted by sex, age smoker and time from symptom onset to care, SLI, ageing index, afro-descendant per 100 inhabitants, indigenous language-speaking per 100 inhabitants, affiliation to health services per 100 inhabitants, members per household, hospitals per 10 000 inhabitants, hospital beds per 10 000 inhabitants. (b) Risk of IMV, model adjusted by sex, age, smoker and time from symptom onset to care, SLI, ageing index, afro-descendant per 100 inhabitants, indigenous language-speaking per 100 inhabitants, affiliation to health services per 100 inhabitants, members per household, hospitals per 10 000 inhabitants, hospital beds per 10 000 inhabitants. (c) Risk of pneumonia, model adjusted by sex, age, smoker and time from symptom onset to care, SLI, ageing index, afro-descendant per 100 inhabitants, indigenous language-speaking per 100 inhabitants, affiliation to health services per 100 inhabitants, members per household, hospitals per 10 000 inhabitants, hospital beds per 10 000 inhabitants. (d) Risk of admission to ICU, model adjusted by sex, age and smoker, SLI, ageing index, afro-descendant per 100 inhabitants, indigenous language-speaking per 100 inhabitants, affiliation to health services per 100 inhabitants, members per household, hospitals per 10 000 inhabitants, hospital beds per 10 000 inhabitants. CKD, chronic kidney disease; COPD, chronic obstructive pulmonary disease; CVD, cardiovascular disease; Immunosupp, immunosuppression; ICU, intensive care unit; IMV, invasive mechanical ventilation.

**Fig. 3.**
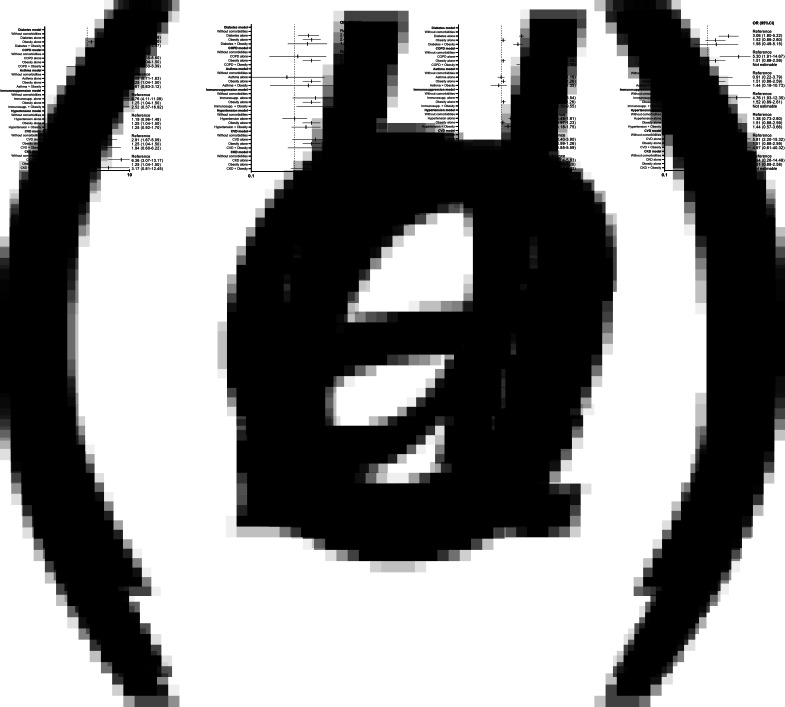
Risk of secondary adverse outcomes in patients with a pending result for SARS-CoV-2 test with obesity or obesity plus one other comorbidity. (a) Risk of hospitalisation, model adjusted by sex, age and time from symptom onset to care, SLI, ageing index, afro-descendant per 100 inhabitants, indigenous language-speaking per 100 inhabitants, affiliation to health services per 100 inhabitants, members per household, hospitals per 10 000 inhabitants, hospital beds per 10 000 inhabitants. (b) Risk of IMV, model adjusted by sex, age and time from symptom onset to care, SLI, ageing index, afro-descendant per 100 inhabitants, indigenous language-speaking per 100 inhabitants, affiliation to health services per 100 inhabitants, members per household, hospitals per 10 000 inhabitants, hospital beds per 10 000 inhabitants. (c) Risk of pneumonia, model adjusted by sex, age and time from symptom onset to care, SLI, ageing index, afro-descendant per 100 inhabitants, indigenous language-speaking per 100 inhabitants, affiliation to health services per 100 inhabitants, members per household, hospitals per 10 000 inhabitants, hospital beds per 10 000 inhabitants. (d) Risk of admission to ICU, model adjusted by sex, age, and time from symptom onset to care, SLI, ageing index, afro-descendant per 100 inhabitants, indigenous language-speaking per 100 inhabitants, affiliation to health services per 100 inhabitants, members per household, hospitals per 10 000 inhabitants, hospital beds per 10 000 inhabitants. CKD, chronic kidney disease; COPD, chronic obstructive pulmonary disease; CVD, cardiovascular disease; Immunosupp, immunosuppression; ICU, intensive care unit; IMV, invasive mechanical ventilation.

Obesity alone and in combination with other comorbidities was a significant risk factor for secondary outcomes (pneumonia, hospitalisation, IMC and ICU admission) in patients positive for SARS-CoV-2 (Fig. 1). However, it was not a determining risk factor for all these endpoints in the negative and pending-result groups (Figs 2 and 3).

## Discussion

The role of obesity as a risk factor for COVID-19-related adverse outcomes is controversial since most studies including largely old, multimorbid and severe-to-critical patients often fail to detect differences in risks [12], but not when including younger patients with lower COVID-19 severity and burden of disease [13]. In this retrospective national cohort study, we sought to study the impact of obesity alone and in combination with other comorbidities as a risk factor for death and other adverse outcomes (hospitalisation, pneumonia, IMV and admission to ICU) in patients evaluated for suspected COVID-19 in ambulatory units and hospitals during the early stage of the pandemic in Mexico.

We observed that obesity was a strong risk factor for mortality and other COVID-19-related adverse outcomes when present alone or in combination with other individual comorbidities; when compared with other individual comorbidities different from obesity, having the strongest association with mortality risk. Increased risk for short-term all-cause mortality and adverse outcomes was greater in patients with a positive test for SARS-CoV-2 with obesity alone or in combination with one accompanying disease. Importantly, in patients with a negative test for SARS-CoV-2 and patients without a definitive result, obesity was not a risk factor for all these outcomes or had a lower increase in risk compared to patients with a positive test.

Our study included both ambulatory and hospitalised patients, with a large representation of COVID-19 patients who did not require hospitalisation (61.1%). SARS-CoV-2-positive patients in our study were younger (46.6, s.d.: 15.5 years) than the majority of studies included in one systematic review to determine mortality risk attributable to comorbidities in patients with COVID-19 [13]; 47.8% of cases in our cohort were between 41 and 60 years old, reflecting that the pandemic affected younger patients in Mexico during its early period. Similar to most other studies, men (57.8%) comprised of a greater proportion of confirmed COVID-19 cases [13]. Hypertension (21.7%), obesity (20.7%) and DM (18.2%) were the most prevalent comorbidities in SARS-CoV-2-positive patients, being higher in non-survivors (43%, 30.5% and 38.8%, respectively). The prevalence of hypertension (18.4%) in people 20 years and older reported in the 2018 National Health and Nutrition Survey is similar to that in our study, whereas numbers for obesity (36.1%) and DM (10.3%) were reported to be higher and lower, respectively, in the Mexican population [7]. Despite being younger, patients in our cohort had a higher prevalence of obesity compared to both survivors (14.2%) and non-survivors (17.1%) included in a systematic review [13].

Variables for adjustment in our study included age, sex, time from symptom onset to medical care, smoking and socio-demographic variables since these have been associated with the risk of developing severe disease and mortality in patients with COVID-19 [10, 20–23]. These covariates have been used in other studies, too [24, 25].

After multivariable adjustment, obesity alone was a strong risk factor for short-term all-cause mortality in patients with a positive test for SARS-CoV-2 (hazard ratio (HR) = 2.47, 95% confidence interval (CI) 2.04–2.98), but not in patients with a negative test (HR = 1.05, 95% CI 0.71–1.53) or with unreleased results (HR = 1.73, 95% CI 0.86–3.48). In the sensitivity analyses, obesity persisted as a strong and significant risk factor for death after excluding patients requiring hospitalisation, IMV or ICU. Obesity alone was also associated with increased risk of secondary adverse outcomes (pneumonia, hospitalisation, IMV and ICU admission). This is consistent with a recent meta-analysis which found that obesity was a significant risk factor for having a positive test for SARS-CoV-2, COVID-19 severity, hospitalisation, IMV, ICU admission and mortality [26].

Other individual comorbidities associated with increased mortality after multivariable adjustment were DM, COPD, immunosuppression, hypertension and CVD, which have also been found as risk factors in other studies [13, 27]. However, obesity alone had larger increases in mortality risk than all these diseases alone, except for immunosuppression. After sensitivity analyses, hypertension, DM and immunosuppression remained as significant risk factors; COPD and CVD alone were not risk factors for death in these analyses, which could be due to the loss of statistical power after the exclusion of patients, although this could reflect that rather than favouring disease progression, COPD and CVD could complicate in-hospital management of patients [28, 29]. Furthermore, distinguishing between current and former smoker statuses is important to adequately assess risks of adverse outcomes in patients with COPD [30]. Similar to other studies, asthma was not a significant risk factor for COVID-19-related adverse outcomes [27, 31]; the combination of asthma and obesity increased mortality risk, but this association was lost after sensitivity analyses. We were not able assess the effect of recent use of systemic corticosteroids in asthmatic patients, which has been shown to be a significant risk factor for adverse outcomes in patients with COVID-19 rather than history of asthma alone [27, 31]. CKD alone did not increase mortality risk, which could be due to the low number of patients with only CKD included in our study since CKD has been found to be a risk factor in others [27, 32]. However, the role of CKD as an individual risk factor for adverse outcomes in COVID-19 should be explored since CKD is often present in combination with DM and hypertension [33], which were individual risk factors in our study.

Obesity combined with DM, immunosuppression and hypertension, had higher increases in mortality risk than in patients with these comorbidities but without obesity. For COPD, asthma, CVD or CKD combined with obesity, mortality risks were not statistically significantly increased, possibly due to the low number of patients presenting these comorbidities combined (*n* = 24, *n* = 84, *n* = 20 and *n* = 11, respectively), which reflects that these comorbidities are often present with more than one concomitant disease in the general population [33–35].

Regarding secondary endpoints (pneumonia, hospitalisation, IMV and ICU admission), obesity alone and in combination with other individual comorbidities was a significant risk factor in patients with a positive test for SARS-CoV-2, but not in those with a negative or pending result.

The main limitation of our study is that obesity was classified as a dichotomous variable in this dataset. Therefore, we were not able to assess if the magnitude of obesity according to different BMI significantly modifies risks. One study found that higher obesity classes gradually increase short-term mortality risk [27]. However, one meta-analysis found that obesity increases the risk of mortality, COVID-19 severity and other adverse outcomes in a non-BMI dependent fashion [26]. Another limitation is that comorbidities could have not been objectively assessed in our study since these were mainly determined through anamnesis. Therefore, we were not able to assess if obesity is an independent risk factor from other comorbidities and potentially confounding unmeasured factors. Other limitations include the retrospective observational limitation of our study, and the unavailability of treatments in the dataset to evaluate their potential impact on mortality risks.

The strengths of our study are its high representativity and generalisability since we included a large sample size from representative COVID-19 ambulatory units and all hospitals in Mexico. Furthermore, we limited potential confounding by highly delimiting the study period and through multivariable adjustment. Finally, this is the first study, to the best of our knowledge, to evaluate risk of death and other adverse outcomes in predominantly young patients who have obesity as their sole comorbidity or in combination with only one additional comorbidity.

This association between obesity and adverse outcomes has been reported in the context of other viral diseases that course with severe pneumonia, such as influenza A H1N1, for which obesity is an independent risk factor for severe disease, hospitalisation, IMV and mortality [36]. Immune dysregulation and disturbances in hormones (i.e. leptin) in patients with obesity have been proposed as the main mechanisms underlying increased susceptibility to influenza viruses [37]. Importantly, obese individuals experience diminished production of type I interferons (IFN) (IFN-*α* and INF-*β*) and delayed pro-inflammatory responses when infected with the influenza A H1N1 virus [38]. This could partly explain increased susceptibility of patients with obesity to SARS-CoV-2 infection since impaired type I IFN responses and altered pro-inflammatory responses are known to occur in patients who experience COVID-19 progression [39].

The mechanical component of patients with obesity may be an essential component that complicates the management of COVID-19. In a multicentre study that included 1317 patients with diabetes, obstructive sleep apnoea (OSA) under treatment was an independent risk factor for 7-day mortality (odds ratio 2.8, 95% CI 1.46–2.38); overweight and obesity were highly prevalent in this study [40]. Furthermore, OSA was a significant risk factor for hospitalisation and respiratory failure after multivariable adjustment in another study [41]. Although risk factors associated with severe-to-critical COVID-19 are similar to those found in the population with the highest prevalence of OSA (men, obesity and advanced age), it is estimated that 90% of patients with OSA are undiagnosed [42].

Upper airway obstruction due to the collapse of pharyngeal soft tissues with excessive accumulation of adipose tissue is associated with intermittent periods of hypoxaemia that affect the pulmonary circulation; up to 70% of patients with OSA have been reported to have pulmonary arterial hypertension (PAH) [43]. Patients with OSA have impaired right ventricular function characterised by decreased ejection fraction and distensibility, without dependency of PAH [44].

Furthermore, it has been shown that, within the pathophysiology of PAH due to OSA, pulmonary microcirculation presents hypoxic vasoconstriction and endothelial dysfunction that promotes a pro-inflammatory and local procoagulant state [45]. These factors may be important in the progression of COVID-19 by promoting viral-induced damage of the endothelium of pulmonary capillaries, causing local inflammation and thrombotic events [46, 47]. Therefore, the combination of these factors could favour severe pulmonary inflammation in patients with obesity, which could explain the greater requirements of IMV, ICU admission and mortality.

From a critical medicine point of view, obesity represents a challenge in critically ill patients requiring IMV since this condition has been reported to generate functional alterations in the respiratory system with reduced compliance of the chest and abdominal walls, airway obstruction, reduction in tele-expiratory volume and a higher incidence of atelectasis [48]. Additionally, obesity is associated with chronic hypoventilation and OSA. As previously mentioned, these conditions increase the risk of difficult ventilation and intubation scenarios during the rapid intubation sequence, as well as alterations in gas exchange, respiratory mechanics and haemodynamic disturbances in patients under IMV, thereby complicating ventilatory management [49]. Patients with obesity who progress to critical COVID-19 present a higher frequency of treatment failure with high-flow nasal cannulas, higher IMV requirements, higher frequencies of ventilatory therapy in the prone position and a higher frequency of retarded removal of IMV and tracheostomies [50].

In conclusion, obesity is a strong risk factor for short-term all-cause mortality and critical illness in Mexican patients with COVID-19 when present as the only comorbidity or in combination with other individual comorbidities. The risk conferred by obesity increases when it is present alongside other comorbidities, particularly, DM, hypertension and immunosuppression.

## Data Availability

The data that support the findings of this study are openly available in Historical COVID-19 Datasets of the Directorate General of Epidemiology of Mexico at https://www.gob.mx/salud/documentos/datos-abiertos-bases-historicas-direccion-general-de-epidemiologia [14].

## Supplementary material

For supplementary material accompanying this paper visit http://dx.doi.org/10.1017/S0950268821001023. click here to view supplementary material
