# Supplementary material for: Obesity is a strong risk factor for short-term mortality and adverse outcomes in Mexican patients with COVID-19: a national observational study
Source: Epidemiol Infect. 2021 Apr 29;149:e109. doi: 10.1017/S0950268821001023 (PMC8134888; doi:10.1017/S0950268821001023)
Supplement: Supplementary file 1 [file hygsup.zip › S0950268821001023sup001.docx]

Epidemiology and Infection

Title: Obesity is a strong risk factor for short-term mortality and adverse outcomes in Mexican patients with COVID-19: A national observational study

Authors: J. M. Vera-Zertuche, J. Mancilla-Galindo, M. Tlalpa-Prisco, P. Aguilar-Alonso, M. M. Aguirre-García, O. Segura-Badilla, M. Lazcano-Hernández, H. I. Rocha-González, A. R. Navarro-Cruz, Kammar-García Ashuin, J. de J. Vidal-Mayo.

**Supplementary Material**

Supplementary Table S1. Baseline and follow-up characteristics of SARS-CoV-2 positive patients with individual comorbidities or obesity plus one other comorbidity.

|  | None  n=8422 | Obesity  n=1599 | DM  n=869 | DM + Obesity  n=292 | COPD  n=75 | COPD + Obesity  n=24 | Asthma  n=249 | Asthma + Obesity  n=84 | Immun  n=97 | Immun+ Obesity  n=26 | HTN  n=1039 | HTN + Obesity  n=479 | CVD  n=90 | CVD + Obesity  n=20 | CKD  n=55 | CKD + Obesity  n=11 |
| --- | --- | --- | --- | --- | --- | --- | --- | --- | --- | --- | --- | --- | --- | --- | --- | --- |
| Sex |  |  |  |  |  |  |  |  |  |  |  |  |  |  |  |  |
| Women, n (%) | 3534 (42) | 679 (42.5) | 314 (36.1) | 120 (41.1) | 21 (28) | 15 (62.5) | 143 (57.4) | 50 (59.5) | 46 (47.4) | 12 (46.2) | 439 (42.3) | 213 (44.5) | 32 (35.6) | 6 (30) | 18 (32.7) | 2 (18.2) |
| Men, n (%) | 4888 (58) | 920 (57.5) | 555 (63.9) | 172 (58.9) | 54 (72) | 9 (37.5) | 106 (42.6) | 34 (40.5) | 51 (52.6) | 14 (53.8) | 600 (57.7) | 266 (55.5) | 58 (64.4) | 14 (70) | 37 (67.3) | 9 (81.8) |
| Age, years | 41.6 (14.3) | 43.1 (11.9) | 54.4 (12.9) | 50.2 (11.5) | 62.5 (16.5) | 58.3 (14.7) | 37.7 (11.2) | 40.1 (11.7) | 44.6 (17.4) | 45.5 (13.3) | 57.2 (14.2) | 52.1 (12.7) | 51.9 (15.9) | 52.6 (16.8) | 46.7 (14.2) | 40.8 (9.1) |
| Smokers, n (%) | 615 (7.3) | 210 (13.1) | 66 (7.6) | 39 (13.4) | 17 (22.7) | 8 (33.3) | 14 (5.6) | 10 (11.9) | 12 (12.4) | 7 (26.9) | 68 (6.5) | 47 (9.8) | 12 (13.3) | 2 (10.0) | 5 (9.1) | 3 (27.3) |
| Pregnancy, n (%) | 71 (0.8) | 10 (0.6) | 2 (0.2) | 1 (03) | 0 (0.0) | 0 (0.0) | 3 (1.2) | 0 (0.0) | 0 (0.0) | 0 (0.0) | 1 (0.1) | 1 (0.2) | 0 (0.0) | 0 (0.0) | 0 (0.0) | 0 (0.0) |
| Time from symptom onset to medical care, days | 4.2 (3.5) | 4.5 (3.2) | 4.5 (3.4) | 4.5 (3.1) | 4.3 (2.8) | 4.3 (2.8) | 4.2 (3.2) | 4.5 (3.1) | 4.3 (3.5) | 3.6 (3.1) | 4.5 (3.5) | 4.5 (3.2) | 4.9 (3.5) | 4.4 (3.9) | 4.4 (3.2) | 3.6 (3.7) |
| Hospitalisation, n (%) | 2296 (27.3) | 662 (41.4) | 532 (61.2) | 169 (57.9) | 43 (57.3) | 15 (62.5) | 47 (18.9) | 21 (25) | 49 (50.5) | 16 (61.5) | 498 (47.9) | 239 (49.9) | 42 (46.7) | 12 (60) | 29 (52.7) | 4 (36.4) |
| Pneumonia, n (%) | 1668 (19.8) | 488 (30.5) | 422 (48.6) | 145 (49.7) | 36 (48) | 10 (41.7) | 41 (16.5) | 20 (23.8) | 39 (40.2) | 11 (42.3) | 401 (38.6) | 194 (40.5) | 31 (34.4) | 7 (35) | 24 (43.6) | 3 (27.3) |
| IMV, n (%) | 214 (2.5) | 77 (4.8) | 57 (6.6) | 24 (8.2) | 6 (8.0) | 2 (8.3) | 3 (1.2) | 2 (2.4) | 4 (4.1) | 0 (0) | 62 (6.0) | 38 (7.9) | 7 (7.8) | 3 (15) | 3 (5.5) | 1 (9.1) |
| ICU admission, n (%) | 235 (2.8) | 81 (5.1) | 56 (6.4) | 20 (6.8) | 7 (9.3) | 2 (8.3) | 4 (1.6) | 2 (2.4) | 6 (6.2) | 1 (3.8) | 63 (6.1) | 38 (7.9) | 8 (8.9) | 1 (5.0) | 3 (5.5) | 0 (0.0) |
| Non-survivors, n (%) | 370 (4.4) | 153 (9.6) | 126 (14.5) | 45 (15.4) | 17 (22.7) | 4 (16.7) | 4 (1.6) | 5 (6.0) | 13 (13.4) | 6 (23.1) | 132 (12.7) | 65 (13.6) | 13 (14.4) | 2 (10.0) | 4 (7.3) | 1 (9.1) |
| Geographical variables | | | | | | | | | | | | | | | | |
| Social lag index | -1.33 (-1.42 –  -1.11) | -1.32 (-1.40 –  -1.11) | -1.29 (-1.38 –  -1.02) | -1.26 (-1.38 –  -1.02) | -1.32 (-1.38 –  -1.02) | -1.20 (-1.33 –  -1.03) | -1.34 (-1.43 –  -1.12) | -1.34 (-1.38 –  -1.19) | -1.34 (-1.44 –  -1.13) | -1.35 (-1.43 –  -1.18) | -1.31 (--1.40 –  -1.13) | -1.32 (-1.38 –  -1.13) | -1.34 (-1.43 –  -1.11) | -1.34 (-1.38 –  -1.04) | -1.29 (-1.40 –  -1.06) | -1.38 (-1.46 –  -0.55) |
| Aging index | 29.1 (21.7-39.7) | 27.8 (21.7-39.6) | 28.8 (22.0-38.2) | 27.9 (16.6-37.8) | 27.7 (20.8-37.8) | 29.7 (23.2-35.7) | 26.9 (21.8-38.2) | 27.3 (22.6-35.9) | 29.3 (22.5-42.5) | 31.1 (23.2-46.1) | 28.6 (22.6-39.6) | 28.7 (22.6-38.2) | 31.9 (22.9-46.4) | 31.1 (22.6-37.8) | 26.3 (23.3-35.6) | 29.4 (18.9-54.2) |
| Afro-descendant | 0.79 (0.09-1.80) | 0.70 (0.08-1.81) | 0.51 (0.07-1.76) | 0.36 (0.08-1.58) | 0.79 (0.08-1.81) | 1.71 (0.06-2.89) | 0.33 (0.04-1.67) | 0.24 (0.06-1.39) | 0.79 (0.05-1.81) | 0.99 (0.04-2.04) | 0.33 (0.04-1.74) | 0.33 (0.04-1.74) | 0.57 (0.07-1.94) | 1.38 (0.15-1.75) | 0.79 (0.06-1.38) | 1.74 (0.23-1.86) |
| Indigenous language-speaking | 1.22 (0.67-1.89) | 1.37 (0.73-2.36) | 1.40 (0.67) | 1.40 (0.67-2.40) | 1.40 (0.73-2.78) | 1.31 (0.53-2.40) | 1.14 (0.63-1.89) | 1.04 (0.63-1.89) | 1.10 (0.41-1.69) | 1.22 (0.83-1.82) | 1.21 (0.63-1.89) | 1.33 (0.63-1.89) | 1.37 (0.75-2.40) | 1.79 (1.26-2.75) | 1.41 (0.50-2.48) | 1.74 (1.37-4.05) |
| Affiliation to health services | 80.5 (77.6-84.2) | 79.6 (76.6-83.8) | 79.8 (76.6-84.3) | 79.6 (76.5-84.3) | 79.5 (75.8-84.6) | 81.5 (76.8-85.0) | 81.7 (78.4-84.3) | 81.7 (78.3-84.3) | 80.6 (77.8-84.3) | 79.5 (76.5-84.1) | 80.7 (77.8-84.3) | 79.6 (77.6-83.8) | 79.7 (77.8-84.9) | 78.6 (74.3-82.9) | 80.6 (77.5-83.5) | 78.6 (77.3-86.7) |
| Members per household | 3.59 (3.45-3.71) | 3.60 (3.5-3.7) | 3.65 (3.50-3.81) | 3.62 (3.50-3.84) | 3.69 (3.53-3.84) | 3.67 (3.46-3.88) | 3.50 (3.41-3.70) | 3.54 (3.41-3.70) | 3.60 (3.45-3.78) | 3.54 (3.44-3.72) | 3.58 (3.45-3.70) | 3.60 (3.49-3.71) | 3.59 (3.45-3.69) | 3.69 (3.53-3.78) | 3.69 (3.58-3.83) | 3.66 (3.54-4.00) |
| Hospitals per 10 000 inhabitants | 3.65 (2.42-5.54) | 3.22 (2.42-5.53) | 3.21 (2.03-5.40) | 3.13 (1.72-5.25) | 3.00 (2.01-5.25) | 2.83 (1.48-4.04) | 3.69 (2.59-6.37) | 3.46 (2.62-6.37) | 3.70 (1.88-5.97) | 3.13 (2.51-5.40) | 3.68 (2.58-6.29) | 3.43 (2.42-5.53) | 4.58 (2.58-6.32) | 2.50 (1.53-5.50) | 3.19 (1.88-5.25) | 4.58 (2.31-8.27) |
| Hospital beds per 10 000 inhabitants | 11.8 (6.86-18.7) | 11.8 (6.86-18.4) | 10.5 (5.5-17.9) | 10.6 (5.2-17.4) | 10.3 (6.1-15.0) | 9.5 (3.5-12.4) | 11.8 (6.9-19.9) | 12.8 (9.3-17.9) | 11.3 (5.6-20.0) | 10.9 (6.6-19.3) | 11.8 (7.9-18.4) | 11.8 (6.9-17.9) | 12.3 (8.2-20.5) | 7.94 (4.2-11.1) | 11.3 (5.5-14.3) | 13.3 (6.3-18.7) |

Data are presented as mean (SD) or median (1Q-3Q).

COPD, chronic obstructive pulmonary disease; CKD, chronic kidney disease; CVD, cardiovascular disease; DM, diabetes mellitus: HTN, hypertension; ICU, intensive care unit; Immun, immunosuppression; IMV, invasive mechanical ventilation
