# Supplementary material for: Obesity is a strong risk factor for short-term mortality and adverse outcomes in Mexican patients with COVID-19: a national observational study
Source: Epidemiol Infect. 2021 Apr 29;149:e109. doi: 10.1017/S0950268821001023 (PMC8134888; doi:10.1017/S0950268821001023)
Supplement: Supplementary file 1 [file hygsup.zip › S0950268821001023sup002.docx]

Epidemiology and Infection

Title: Obesity is a strong risk factor for short-term mortality and adverse outcomes in Mexican patients with COVID-19: A national observational study

Authors: J. M. Vera-Zertuche, J. Mancilla-Galindo, M. Tlalpa-Prisco, P. Aguilar-Alonso, M. M. Aguirre-García, O. Segura-Badilla, M. Lazcano-Hernández, H. I. Rocha-González, A. R. Navarro-Cruz, Kammar-García Ashuin, J. de J. Vidal-Mayo.

**Supplementary Material**

Supplementary Table S2. Baseline and follow-up characteristics of SARS-CoV-2 negative patients with individual comorbidities or obesity plus one other comorbidity.

|  | None  n= 30016 | Obesity  n= 3362 | DM  n=1390 | DM + Obesity  n=389 | COPD  n=347 | COPD + Obesity  n=45 | Asthma  n=1557 | Asthma + Obesity  n=314 | Immun  n=639 | Immun+ Obesity  n=63 | HTN  n=2354 | HTN + Obesity  n=922 | CVD  n=389 | CVD + Obesity  n=70 | CKD  n=182 | CKD + Obesity  n=27 |
| --- | --- | --- | --- | --- | --- | --- | --- | --- | --- | --- | --- | --- | --- | --- | --- | --- |
| Sex |  |  |  |  |  |  |  |  |  |  |  |  |  |  |  |  |
| Women, n (%) | 15994 (53.3) | 1831 (54.5) | 715 (51.4) | 234 (60.2) | 165 (47.6) | 24 (53.3) | 929 (59.7) | 205 (65.3) | 347 (54.3) | 36 (57.1) | 1146 (48.7) | 514 (55.7) | 199 (51.2) | 32 (45.7) | 76 (41.8) | 14 (51.9) |
| Men, n (%) | 14022 (46.7) | 1531 (45.5) | 675 (48.6) | 155 (39.8) | 182 (52.4) | 21 (46.7) | 628 (40.3) | 109 (34.7) | 292 (45.7) | 27 (42.9) | 1208 (51.3) | 408 (44.3) | 190 (48.8) | 38 (54.3) | 106 (58.2) | 13 (48.1) |
| Age, years | 34.5 (15.6) | 38.9 (12.2) | 50.8 (14.5) | 48.4 (11.9) | 61.7 (20.0) | 59.9 (18.4) | 32.5 (13.8) | 37.1 (11.9) | 36.6 (19.7) | 44.0 (15.9) | 53.8 (15.7) | 48.9 (13.5) | 40.9 (24.5) | 46.9 (17.8) | 43.5 (20.9) | 44.1 (13.9) |
| Smokers, n (%) | 2542 (8.5) | 601 (17.9) | 145 (10.4) | 70 (18) | 90 (25.9) | 14 (31.1) | 134 (8.6) | 40 (12.7) | 53 (8.3) | 8 (12.7) | 233 (9.9) | 126 (13.7) | 41 (10.5) | 16 (22.9) | 24 (13.2) | 7 (25.9) |
| Pregnancy, n (%) | 522 (1.7) | 41 (1.2) | 9 (0.6) | 5 (1.3) | 1 (0.3) | 0 (0.0) | 27 (1.7) | 7 (2.2) | 8 (1.3) | 1 (1.6) | 7 (0.3) | 4 (0.4) | 4 (1.0) | 0 (0.0) | 2 (1.1) | 0 (0.0) |
| Time from symptom onset to medical care, days | 3.4 (3.4) | 3.6 (3.3) | 3.6 (4.0) | 3.4 (3.8) | 3.2 (3.4) | 3.3 (2.9) | 3.6 (3.6) | 4.1 (4.0) | 2.8 (2.9) | 2.9 (3.1) | 3.5 (3.8) | 3.7 (3.3) | 3.3 (3.2) | 3.6 (4.6) | 3.0 (3.2) | 3.7 (2.8) |
| Hospitalisation, n (%) | 4270 (14.2) | 512 (15.2) | 511 (36.8) | 116 (29.8) | 186 (53.6) | 26 (57.8) | 231 (14.8) | 53 (16.9) | 319 (49.9) | 29 (46.0) | 553 (23.5) | 209 (22.7) | 154 (39.6) | 21 (30.0) | 82 (45.1) | 10 (37.0) |
| Pneumonia, n (%) | 2648 (8.8) | 335 (10.0) | 342 (24.6) | 80 (20.6) | 128 (36.9) | 15 (33.3) | 133 (8.5) | 23 (7.3) | 195 (30.5) | 22 (34.9) | 384 (16.3) | 127 (13.8) | 90 (23.1) | 18 (25.7) | 56 (30.8) | 4 (14.8) |
| IMV, n (%) | 226 (0.8) | 33 (1.0) | 49 (3.5) | 13 (3.3) | 8 (2.3) | 2 (4.4) | 7 (0.4) | 2 (0.6) | 26 (4.1) | 2 (3.2) | 31 (1.3) | 15 (1.6) | 12 (3.1) | 2 (2.9) | 4 (2.2) | 0 (0.0) |
| ICU admission, n (%) | 301 (1.0) | 45 (1.3) | 52 (3.7) | 14 (3.6) | 12 (3.5) | 3 (6.7) | 10 (0.6) | 1 (0.3) | 28 (4.4) | 7 (11.1) | 48 (2.0) | 14 (1.5) | 10 (2.6) | 0 (0.0) | 6 (3.3) | 1 (3.7) |
| Non-survivors, n (%) | 230 (0.8) | 30 (0.9) | 61 (4.4) | 11 (2.8) | 31 (8.9) | 2 (4.4) | 6 (0.4) | 4 (1.3) | 29 (4.5) | 3 (4.8) | 59 (2.5) | 17 (1.8) | 14 (3.6) | 4 (5.7) | 9 (4.9) | 2 (7.4) |
| Geographical variables | | | | | | | | | | | | | | | | |
| Social lag index | -1.29 (-1.42 –  -1.07) | -1.29 (-1.40 –  -1.05) | -1.26 (-1.41 –  -0.98) | -1.26 (-1.37 –  -0.98) | -1.26 (-1.37 –  -0.89) | -1.17 (-1.36 –  -0.86) | -1.31 (-1.42 –  -1.09) | -1.31 (-1.43 –  -1.06) | -1.27 (-1.42 –  -1.03) | -1.31 (-1.39 –  -1.11) | -1.29 (-1.41 –  -1.08) | -1.29 (-1.38 –  -1.06) | -1.29 (-1.41 –  -1.04) | -1.24 (-1.43 –  0.83) | -1.27 (-1.42 –  -1.01) | -1.24 (-1.40 –  -0.92) |
| Aging index | 28.6 (21.0-38.6) | 28.6 (21.8-38.2) | 29.1 (22.3-38.6) | 27.9 (21.0-37.9) | 27.7 (21.8-37.8) | 31.7 (23.1-36.9) | 29.3 (22.3-41.6) | 28.6 (21.5-41.6) | 28.7 (21.5-39.6) | 27.7 (20.9-38.6) | 29.1 (21.8-38.6) | 27.9 (21.3-35.9) | 27.9 (21.7-39.7) | 29.1 (21.1-40.1) | 28.9 (21.1-37.8) | 30.2 (22.9-46.0) |
| Afro-descendant | 0.23 (0.04-1.39) | 0.23 (0.03-1.47) | 0.28 (0.02-1.41) | 0.20 (0.01-1.44) | 0.10 (0.01-1.20) | 0.11 (0.00-1.19) | 0.23 (0.04-1.47) | 0.23 (0.06-1.19) | 0.23 (0.03-1.39) | 0.20 (0.02-1.54) | 0.23 (0.03-1.38) | 0.20 (0.03-1.40) | 0.15 (0.01-1.44) | 0.27 (0.02-1.49) | 0.10 (0.02-1.19) | 0.02 (0.00-1.81) |
| Indigenous language-speaking | 0.96 (0.00-1.72) | 1.00 (0.00-1.79) | 1.00 (0.00-1.79) | 1.00 (0.00-1.79) | 0.89 (0.00-1.79) | 1.00 (0.00-1.79) | 1.00 (0.21-1.74) | 0.95 (0.23-1.53) | 0.96 (0.00-1.70) | 0.90 (0.00-1.79) | 0.96 (0.21-1.74) | 0.96 (0.00-1.79) | 0.80 (0.00-1.46) | 0.95 (0.00-1.46) | 0.94 (0.00-1.78) | 0.96 (0.00-1.91) |
| Affiliation to health services | 82.6 (78.6-86.0) | 82.3 (78.6-86.0) | 82.4 (78.4-86.2) | 82.3 (78.4-86.9) | 82.8 (78.4-86.1) | 82.0 (79.2-86.9) | 82.4 (78.6-86.1) | 82.9 (79.0-86.3) | 82.4 (78.4-86.0) | 82.3 (78.6-85.4) | 82.6 (78.6-86.0) | 82.6 (78.4-86.1) | 82.4 (79.0-86.0) | 79.6 (77.1-85.7) | 82.5 (78.6-86.0) | 82.3 (77.4-88.1) |
| Members per household | 3.66 (3.50-3.80) | 3.65 (3.50-3.80) | 3.66 (3.50-3.80) | 3.68 (3.51-3.84) | 3.70 (3.53-3.84) | 3.71 (3.64-3.88) | 3.63 (3.49-3.74) | 3.59 (3.5-3.7) | 3.68 (3.53-3.81) | 3.68 (3.50-3.80) | 3.65 (3.50-3.78) | 3.65 (3.50-3.79) | 3.66 (3.50-3.80) | 3.68 (3.55-3.82) | 3.70 (3.56-3.82) | 3.64 (3.49-3.84) |
| Hospitals per 10 000 inhabitants | 4.16 (2.31-6.30) | 3.83 (2.31-5.54) | 3.62 (2.03-5.59) | 3.43 (1.75-5.25) | 4.16 (2.17-5.49) | 3.21 (1.71-4.86) | 4.28 (2.31-6.37) | 4.2 (2.6-6.3) | 3.95 (2.34-3.60) | 3.57 (2.42-6.37) | 4.16 (2.42-6.30) | 3.62 (2.31-5.51) | 3.76 (2.38-5.53) | 4.04 (2.34-8.21) | 3.57 (2.02-6.27) | 2.72 (0.00-5.01) |
| Hospital beds per 10 000 inhabitants | 11.8 (6.9-18.5) | 11.8 (6.8-18.4) | 11.4 (5.3-18.4) | 10.9 (5.2-17.7) | 11.6 (5.9-18.4) | 10.7 (5.5-15.7) | 13.0 (7.6-20.5) | 12.5 (7.9-20.5) | 11.8 (6.7-19.3) | 11.6 (7.5-17.2) | 11.8 (6.9-18.7) | 11.6 (6.6-17.9) | 11.9 (6.6-19.3) | 12.4 (5.01-19.4) | 11.6 (6.0-17.2) | 11.4 (0.0-17.4) |

Data are presented as mean (SD) or median (1Q-3Q).

COPD, chronic obstructive pulmonary disease; CKD, chronic kidney disease; CVD, cardiovascular disease; DM, diabetes mellitus: HTN, hypertension; ICU, intensive care unit; Immun, immunosuppression; IMV, invasive mechanical ventilation
