# Supplementary material for: Obesity is a strong risk factor for short-term mortality and adverse outcomes in Mexican patients with COVID-19: a national observational study
Source: Epidemiol Infect. 2021 Apr 29;149:e109. doi: 10.1017/S0950268821001023 (PMC8134888; doi:10.1017/S0950268821001023)
Supplement: Supplementary file 1 [file hygsup.zip › S0950268821001023sup003.docx]

Epidemiology and Infection

Title: Obesity is a strong risk factor for short-term mortality and adverse outcomes in Mexican patients with COVID-19: A national observational study

Authors: J. M. Vera-Zertuche, J. Mancilla-Galindo, M. Tlalpa-Prisco, P. Aguilar-Alonso, M. M. Aguirre-García, O. Segura-Badilla, M. Lazcano-Hernández, H. I. Rocha-González, A. R. Navarro-Cruz, Kammar-García Ashuin, J. de J. Vidal-Mayo.

**Supplementary Material**

Supplementary Table S3. Baseline and follow-up characteristics of SARS-CoV-2 pending-result patients with individual comorbidities or obesity plus one other comorbidity.

|  | None  n=5057 | Obesity  n= 774 | DM  n=412 | DM + Obesity  n=121 | COPD  n=52 | COPD + Obesity  n=14 | Asthma  n=180 | Asthma + Obesity  n=54 | Immun  n=77 | Immun+ Obesity  n=5 | HTN  n=492 | HTN + Obesity  n=212 | CVD  n=63 | CVD + Obesity  n=14 | CKD  n=36 | CKD + Obesity  n=9 |
| --- | --- | --- | --- | --- | --- | --- | --- | --- | --- | --- | --- | --- | --- | --- | --- | --- |
| Sex |  |  |  |  |  |  |  |  |  |  |  |  |  |  |  |  |
| Women, n (%) | 2323 (45.9) | 378 (48.8) | 163 (39.6) | 59 (48.8) | 21 (40.4) | 4 (28.6) | 99 (55.0) | 34 (63.0) | 40 (51.9) | 2 (40.0) | 199 (40.4) | 94 (44.3) | 31 (49.2) | 8 (57.1) | 14 (38.9) | 2 (22.2) |
| Men, n (%) | 2734 (54.1) | 396 (51.2) | 249 (60.4) | 62 (51.2) | 31 (59.6) | 10 (71.4) | 81 (45.0) | 20 (37.0) | 37 (48.1) | 3 (60.0) | 293 (59.6) | 118 (55.7) | 32 (50.8) | 6 (42.9) | 22 (61.1) | 7 (77.8) |
| Age, years | 38.1 (15.7) | 41.7 (11.9) | 53.1 (13.6) | 48.1 (11.3) | 62.4 (19.9) | 55.4 (14.4) | 31.6 (13.8) | 36.4 (11.7) | 35.5 (19.7) | 49.0 (6.0) | 54.7 (15.3) | 51.0 (12.5) | 41.1 (24.1) | 42.5 (16.9) | 42.6 (21.0) | 49.7 (11.4) |
| Smokers, n (%) | 385 (7.6) | 116 (15.0) | 32 (7.8) | 17 (14.0) | 10 (19.2) | 5 (35.7) | 13 (7.2) | 11 (20.4) | 5 (6.5) | 1 (20.0) | 47 (9.6) | 22 (10.4) | 2 (3.2) | 1 (7.1) | 7 (19.4) | 2 (22.2) |
| Pregnancy, n (%) | 46 (0.9) | 5 (0.6) | 1 (0.2) | 0 (0.0) | 0 (0.0) | 0 (0.0) | 2 (1.1) | 0 (0.0) | 1 (1.3) | 0 (0.0) | 1 (0.2) | 1 (0.5) | 0 (0.0) | 0 (0.0) | 0 (0.0) | 0 (0.0) |
| Time from symptom onset to medical care, days | 3.4 (3.2) | 3.7 (2.8) | 3.8 (2.9) | 4.1 (3.1) | 4.3 (3.9) | 5.4 (3.1) | 3.5 (2.8) | 3.5 (2.8) | 3.7 (3.5) | 3.8 (2.4) | 3.8 (2.9) | 4.3 (3.7) | 4.5 (3.9) | 5.8 (5.7) | 2.7 (2.9) | 4.1 (2.9) |
| Hospitalisation, n (%) | 1055 (20.9) | 205 (26.5) | 229 (55.6) | 66 (54.5) | 36 (69.2) | 5 (35.7) | 30 (16.7) | 13 (24.1) | 44 (57.1) | 3 (60) | 179 (36.4) | 74 (34.9) | 27 (42.9) | 5 (35.7) | 21 (58.3) | 5 (55.6) |
| Pneumonia, n (%) | 717 (14.2) | 165 (21.3) | 178 (43.2) | 48 (39.7) | 26 (50.0) | 5 (35.7) | 18 (10.0) | 9 (16.7) | 29 (37.7) | 2 (40.0) | 127 (25.8) | 65 (30.7) | 14 (22.2) | 3 (21.4) | 16 (44.4) | 4 (44.4) |
| IMV, n (%) | 65 (1.3) | 25 (3.2) | 18 (4.4) | 4 (3.3) | 2 (3.8) | 0 (0.0) | 1 (0.6) | 1 (1.9) | 3 (3.9) | 1 (20.0) | 12 (2.4) | 8 (3.8) | 3 (4.8) | 1 (7.1) | 0 (0.0) | 1 (11.1) |
| ICU admission, n (%) | 21 (5.1) | 17 (2.2) | 21 (5.1) | 3 (2.5) | 5 (9.6) | 0 (0.0) | 2 (1.1) | 1 (1.9) | 5 (6.5) | 0 (0.0) | 13 (2.6) | 5 (2.4) | 5 (7.9) | 1 (7.1) | 1 (2.8) | 0 (0.0) |
| Non-survivors, n (%) | 39 (0.8) | 10 (1.3) | 10 (2.4) | 2 (1.7) | 1 (1.9) | 1 (7.1) | 1 (0.6) | 0 (0.0) | 3 (3.9) | 1 (20.0) | 10 (2.0) | 4 (1.9) | 2 (3.2) | 1 (7.1) | 2 (5.6) | 1 (11.1) |
| Geographical variables | | | | | | | | | | | | | | | | |
| Social lag index | -1.29 (-1.42 –  -1.01) | -1.28 (-1.38 –  -1.02) | -1.24 (-1.36 –  -0.95) | -1.34 (-1.38 –  -0.99) | -1.13 (-1.32 –  -0.89) | -1.25 (-1.35 –  -0.79) | -1.27 (-1.38 –  -0.99) | -1.29 (-1.36 –  -1.02) | -1.24 (-1.37 –  -0.98) | -1.26 (-1.47 –  -0.79) | -1.27 (-1.39 –  -1.01) | -1.29 (-1.36 –  -1.02) | -1.29 (-1.37 –  -1.06) | 1.06 (-1.35 – -0.81) | -1.26 (-1.43 –  -0.88) | -1.33 (-1.57 –  -0.88) |
| Aging index | 29.3 (21.5-39.6) | 28.5 (21.5-38.6) | 28.1 (19.6-35.7) | 30.1 (21.9-40.7) | 28.9 (22.2-35.2) | 28.8 (20.6-31.4) | 29.4 (21.6-36.4) | 29.3 (21.5-37.8) | 28.4 (20.9-32.5) | 19.9 (16.3-44.7) | 29.3 (21.5-38.6) | 27.3 (20.9-35.2) | 27.6 (21.8-38.8) | 26.9 (21.8-39.6) | 30.6 (15.3-43.6) | 35.2 (17.5-75.5) |
| Afro-descendant | 0.44 (0.03-1.74) | 0.61 (0.04-1.76) | 0.33 (0.03-1.74) | 0.79 (0.05-1.80) | 0.07 (0.00-1.33) | 0.10 (0.00-1.19) | 0.20 (0.02-1.29) | 0.20 (0.00-1.39) | 0.23 (0.00-1.75) | 1.38 (0.20-2.59) | 0.44 (0.03-1.76) | 0.33 (0.02-1.74) | 0.33 (0.03-1.54) | 0.36 (0.02-1.47) | 0.61 (0.10-1.52) | 1.76 (0.21-2.66) |
| Indigenous language-speaking | 1.10 (0.00-1.79) | 1.14 (0.00-1.89) | 1.22 (0.00-2.40) | 1.26 (0.63-1.79) | 0.50 (0.00-1.78) | 0.91 (0.64-2.02) | 0.95 (0.00-1.73) | 0.94 (0.00-1.79) | 1.21 (0.29-2.00) | 0.95 (0.45-2.41) | 1.14 (0.32-2.11) | 1.21 (0.32-1.91) | 1.22 (0.29-1.89) | 1.11 (0.00-2.80) | 0.98 (0.00-2.55) | 1.22 (1.02-2.29) |
| Affiliation to health services | 81.3 (77.4-85.5) | 81.1 (76.6-85.3) | 80.1 (75.6-85.5) | 79.6 (75.8-84.8) | 82.3 (76.6-86.1) | 83.0 (80.6-87.8) | 81.7 (77.5-86.1) | 82.5 (78.3-86.5) | 82.3 (76.3-86.4) | 75.6 (61.6-78.4) | 81.0 (76.8-84.5) | 80.2 (76.3-84.3) | 82.0 (77.8-84.4) | 84.6 (75.7-89.9) | 83.7 (79.4-85.9) | 79.5 (76.6-82.5) |
| Members per household | 3.68 (3.53-3.84) | 3.69 (3.50-3.85) | 3.69 (3.53-3.88) | 3.65 (3.45-3.83) | 3.80 (3.66-4.05) | 3.5 (3.5-3.8) | 3.61 (3.48-3.80) | 3.52 (3.45-3.70) | 3.68 (3.51-3.86) | 3.69 (3.49-3.91) | 3.65 (3.49-3.82) | 3.70 (3.50-3.86) | 3.71 (3.54-3.90) | 3.72 (3.64-3.91) | 3.68 (3.51-3.79) | 3.65 (3.03-3.92) |
| Hospitals per 10 000 inhabitants | 3.51 (2.17-5.51) | 3.21 (1.72-5.32) | 3.00 (1.72-5.01) | 2.82 (1.79-5.29) | 2.58 (0.54-4.64) | 5.07 (2.57-5.59) | 3.64 (1.97-5.58) | 3.00 (0.90-5.59) | 3.21 (1.64-5.04) | 3.00 (1.78-5.69) | 3.62 (1.99-5.40) | 3.21 (2.31-5.47) | 3.43 (2.42-5.51) | 3.03 (2.42-4.37) | 3.65 (1.33-8.02) | 5.10 (2.82-9.75) |
| Hospital beds per 10 000 inhabitants | 11.3 (5.6-18.5) | 10.7 (5.1-17.0) | 9.3 (4.5-15.0) | 9.6 (4.9-20.5) | 6.9 (2.3-12.8) | 10.1 (6.8-14.1) | 11.7 (5.3-18.5) | 11.4 (2.5-16.6) | 9.9 (4.15.2) | 10.8 (5.8-16.7) | 10.8 (5.3-17.9) | 10.5 (6.8-15.7) | 12.3 (6.9-16.5) | 8.3 (5.3-14.8) | 11.6 (4.8-20.3) | 14.3 (9.9-28.8) |

Data are presented as mean (SD) or median (1Q-3Q).

COPD, chronic obstructive pulmonary disease; CKD, chronic kidney disease; CVD, cardiovascular disease; DM, diabetes mellitus: HTN, hypertension; ICU, intensive care unit; Immun, immunosuppression; IMV, invasive mechanical ventilation
